# Physicians’ Visiting List

**Published:** 1885-12

**Authors:** 


**Physician's Visiting List, 1886.** Philadelphia: Lindsay & Blakiston, 1012 Walnut street.

The present is the thirty-fifth year of its publication, and, in spite of many rivals, it holds its own against all comers. It contains calendar, list of poisons and antidotes, dose tables re-written in accordance with the sixth revision of the U. S. Pharmacopœia, Marshall Hall's Ready Method in Asphyxia, lists of new remedies, Sylvester's method for producing artificial respiration, with illustrations; diagram for diagnosing diseases of heart, lungs, etc.

The quality of the leather used in binding this list has been again improved, and a superior pencil, with nickel tip, manufactured especially for it, has been added.

For sizes and prices, see advertisement in our advertising columns.

